# Response of the root morphological structure of *Fokienia hodginsii* seedlings to competition from neighboring plants in a heterogeneous nutrient environment

**DOI:** 10.3389/fpls.2023.1327322

**Published:** 2024-01-17

**Authors:** Bingjun Li, Mi Deng, Yanmei Pan, Wenchen Chen, Tianyou He, Liguang Chen, Yushan Zheng, Jundong Rong

**Affiliations:** ^1^ College of Forestry, Fujian Agriculture and Forestry University, Fuzhou, China; ^2^ College of Landscape Architecture and Art, Fujian Agriculture and Forestry University, Fuzhou, China

**Keywords:** *Fokienia hodginsii*, heterogeneous nutrient environment, planting pattern, root morphology, anatomical structure

## Abstract

**Introduction:**

Critical changes often occur in *Fokienia hodginsii* seedlings during the process of growth owing to differences in the surrounding environment. The most common differences are heterogeneous nutrient environments and competition from neighboring plants.

**Methods:**

In this study, we selected one-year-old, high-quality *Fokienia hodginsii* seedlings as experimental materials. Three planting patterns were established to simulate different competitive treatments, and seedlings were also exposed to three heterogeneous nutrient environments and a homogeneous nutrient environment (control) to determine their effect on the root morphology and structure of *F. hodginsii* seedlings.

**Results:**

Heterogeneous nutrient environments, compared with a homogeneous environment, significantly increased the dry matter accumulation and root morphology indexes of the root system of *F. hodginsii*, which proliferated in nutrient-rich patches, and the P heterogeneous environment had the most significant enhancement effect, with dry matter accumulation 70.2%, 7.0%, and 27.0% higher than that in homogeneous and N and K heterogeneous environments, respectively. Homogeneous environments significantly increased the specific root length and root area of the root system; the dry matter mass and morphological structure of the root system of *F*. *hodginsii* with a heterospecific neighbor were higher than those under conspecific neighbor and single-plant treatments, and the root area of the root system under the conspecific neighbor treatment was higher than that under the heterospecific neighbor treatment, by 20% and 23%, respectively. Moreover, the root system under heterospecific neighbor treatment had high sensitivity; the heterogeneous nutrient environment increased the mean diameter of the fine roots of the seedlings of *F. hodginsii* and the diameter of the vascular bundle, and the effect was most significant in the P heterogeneous environment, exceeding that in the N and K heterogeneous environments. The effect was most significant in the P heterogeneous environment, which increased fine root diameter by 20.5% and 10.3%, respectively, compared with the homogeneous environment; in contrast, the fine root vascular ratio was highest in the homogeneous environment, and most of the indicators of the fine root anatomical structure in the nutrient-rich patches were of greater values than those in the nutrient-poor patches in the different heterogeneous environments; competition promoted most of the indicators of the fine root anatomical structure of *F. hodginsii* seedlings. According a principal component analysis (PCA), the N, Pm and K heterogeneous environments with heterospecific neighbors and the P heterogeneous environment with a conspecific neighbor had higher evaluation in the calculation of eigenvalues of the PCA.

**Discussion:**

The root dry matter accumulation, root morphology, and anatomical structure of *F*. *hodginsii* seedlings in the heterogeneous nutrient environment were more developed than those in the homogeneous nutrient environment. The effect of the P heterogeneous environment was the most significant. The heterospecific neighbor treatment was more conducive to the expansion and development of root morphology of *F. hodginsii* seedlings than were the conspecific neighbor and single-plant treatments.

## 1 Introduction

The nutrients in forest soil are highly heterogeneously distributed (Liu et al., 2014). Plants generate a series of plastic, morphological responses during their growth and development to adapt to the environment and more fully acquire resources. These responses include root elongation, an increase in the root surface area and lateral roots, alterations in their microstructure (Li et al., 2012; Walk et al., 2014; Zou et al., 2014), and an enhancement in the ability to absorb highly mobile ions, such as nitrate (NO_3_^-^) (Wang et al., 2013). Compared with a homogeneous nutrient environment, plants typically obtain more nutrient resources in heterogeneous nutrient environments (Ma et al., 2009; Zhang et al., 2012). Root morphology is commonly a sensitive indicator for plants in heterogeneous nutrient environments; it affects plant growth, development, and relative competitiveness, thereby affecting the community composition, structure, and productivity (Rajaniemi, 2007; Mommer et al., 2011). Soil heterogeneity can result in varied root forms in different nutrient patches. It has been shown that when *Pinus massoniana* encounter heterogeneous nutrient patches, they display higher root dry matter mass, total root length, root surface area, and root volume than when in homogeneous nutrient environments (Ma et al., 2010). This results in a broader root distribution, higher foraging accuracy, and sensitivity to responses, as well as root morphology plasticity. A study on the tea family (Theaceae) member *Schima superba* also showed that the total root length, total surface area, and total volume in heterogeneous environments increased significantly, which indicated that soil heterogeneity has varying degrees of impact on plant root morphology (Yao et al., 2017). In recent years, the response of plants to the spatial heterogeneity of available soil resources and the response of plant roots to heterogeneous nutrients have gradually become an active area of forestry research. Currently, most research has focused on comparisons of root morphology and nutrient absorption efficiency (Schmid et al., 2015; Novoplansky, 2009), and there has been minimal research on the impact of heterogeneous environments on plant root morphology from the perspective of microanatomical structure. Few studies have been conducted on the impact of nutrient heterogeneity on the anatomical structure of plant roots.

Competition is an important phenomenon during the process of plant growth (Chen et al., 2015; Ren and Yang, 2016). Plant roots are inevitably influenced by neighboring plant roots during the process of exploring heterogeneously distributed nutrients. The plastic response of plants to nutrients and neighboring plant competition play important roles in the efficient utilization of nutrient resources and the improvement of productivity (Rolo and Moreno, 2012). Plants can utilize root morphological plasticity to increase their competitiveness for foraging nutrient and water resources, thereby influencing competitive relationships between neighboring plants (Kigathi et al., 2013). Therefore, plants with high morphological plasticity can quickly occupy resources and obtain more water and nutrition resources. This consequently promotes their growth, which results in competitive advantages. Additionally, plants undergo adjustments in their morphological plasticity during their process of growth, which results in the differentiation of ecological niches (Hodge, 2004). This enables different plant individuals to utilize nutrient resources from different ecological regions, which reduces the competition for resources and enables species to coexist. Compared with herbaceous plants, forest trees encounter more nutrient patches and perceive a larger scale of such patches during their growth (Wu et al., 2008; Flatscher et al., 2012; Mou et al., 2013). Plants are frequently confronted by diverse and heterogeneous soil environments during the process of growth and development in addition to different types of patterns of neighboring plant competition; these two factors interact with each other to regulate plant growth (Mommer et al., 2012; Wu et al., 2014). Current studies have rarely examined the relationship between neighboring plant competition and foraging behavior. Therefore, the comprehensive impact and regulatory mechanism of planting patterns and heterogeneous nutrient patches on plant root morphological structure and function have also been a highly active research topic in recent years.

Fujian cypress (*Fokienia hodginsii* [Dunn] Henry et Thomas) is a second-class key protected wild plant species in China. It is tolerant of shade in its juvenile years and suitable for planting in slightly acidic to acidic yellow soil and yellow-brown soil. It features shallow roots, resistance to drought and infertile soil, developed lateral roots, and no obvious main roots. It is primarily distributed in forest areas with an altitude of 350–700 m in southwestern, southern, and eastern China (Li et al., 2022). In recent years, studies on *F. hodginsii* have concentrated mainly on cultivation techniques and artificial forest management (Muhammad et al., 2020; Li et al., 2023). However, in these studies, the soil nutrients at the seedling rearing stage have been mostly homogeneous, and a single-plant pattern was the main planting pattern, which renders this body of research unable to assess the adaptability and competitiveness of *F. hodginsii* seedlings to nutrient environments in terms of nutrient heterogeneity and interspecies competition. Some previous studies have also demonstrated that different tree species show significant differences in their response to heterogeneous nutrient environments and competition from neighboring plants (Wang et al., 2007; Ma et al., 2010), but there is a lack of relevant research on *F*. *hodginsii*. Therefore, in this study, one-year-old *F. hodginsii* seedlings were selected, which were assumed to normally grow in different heterogeneous nutrient environments and encounter different types of neighboring plant competition, to determine whether the root morphological structure of *F. hodginsii* seedlings varied according to the nutrient environment and the planting pattern. Referring to the previous studies, nitrogen (N), phosphorous (P), and potassium (K) heterogeneous nutrient environments were set up and compared with the homogeneous environments. N, P, and K are the dominant nutrient elements in soils, and their distributions are most commonly heterogeneous in soils. In this work, neighbor competition was divided into interspecific competition and intraspecific competition, using single-plant, conspecific neighbor, and heterospecific neighbor planting patterns. The results of this study provide a reference method for the selection of robust Fujian cypress varieties and an empirical basis for better adaptation of Fujian cypress seedlings to heterogeneous nutrient environments and neighboring plant competition during the early stage of afforestation work.

## 2 Materials and methods

### 2.1 Overview of the experimental site

The experiment was conducted in the greenhouse facilities (119°13′51.18″E, 26°05′4.35″N) of the College of Landscape Architecture and Art, Fujian Agriculture and Forestry University (Fuzhou, China). The greenhouse was built for experimental (educational) use, and it was thoroughly ventilated, cooled with sprays of water, and shaded from the sun by a net. During the experiment, the average temperature of the greenhouse was between 18°C and 28°C,; the relative humidity was higher than 78%, the daylight was from approximately 6:00 to 18:00, and the average duration of sunshine was approximately 12 h.

### 2.2 Experimental materials

The experiment used one-year-old *F*. *hodginsii* seedlings, from robust families cultivated in the state-owned Fengtian Forest Farm in Anxi County, Quanzhou City, Fujian Province, China. To ensure the consistent initial state of the experimental materials, 180 F. *hodginsii* plants were selected. They had an average ground diameter of 2.65 ± 0.86 mm and an average height of 21.47 ± 4.12 cm. Pot experiments were conducted with the establishment of four heterogeneous and homogeneous nutrient environments using nitrogen (N), phosphorus (P), and potassium (K). The pot culture medium was collected from barren acidic red soil on the back hill of Fujian Agricultural and Forestry University with an organic matter content of 5.91 g·kg^-1^ and total N and total P contents of 0.43 and 0.40 g·kg^-1^, respectively. The contents of hydrolyzed N, available K, and available P were 29.08, 238.68, and 6.15 mg· kg^-1^, respectively, and the soil had a pH of 4.97.

### 2.3 Experimental design

**Figure 1:**
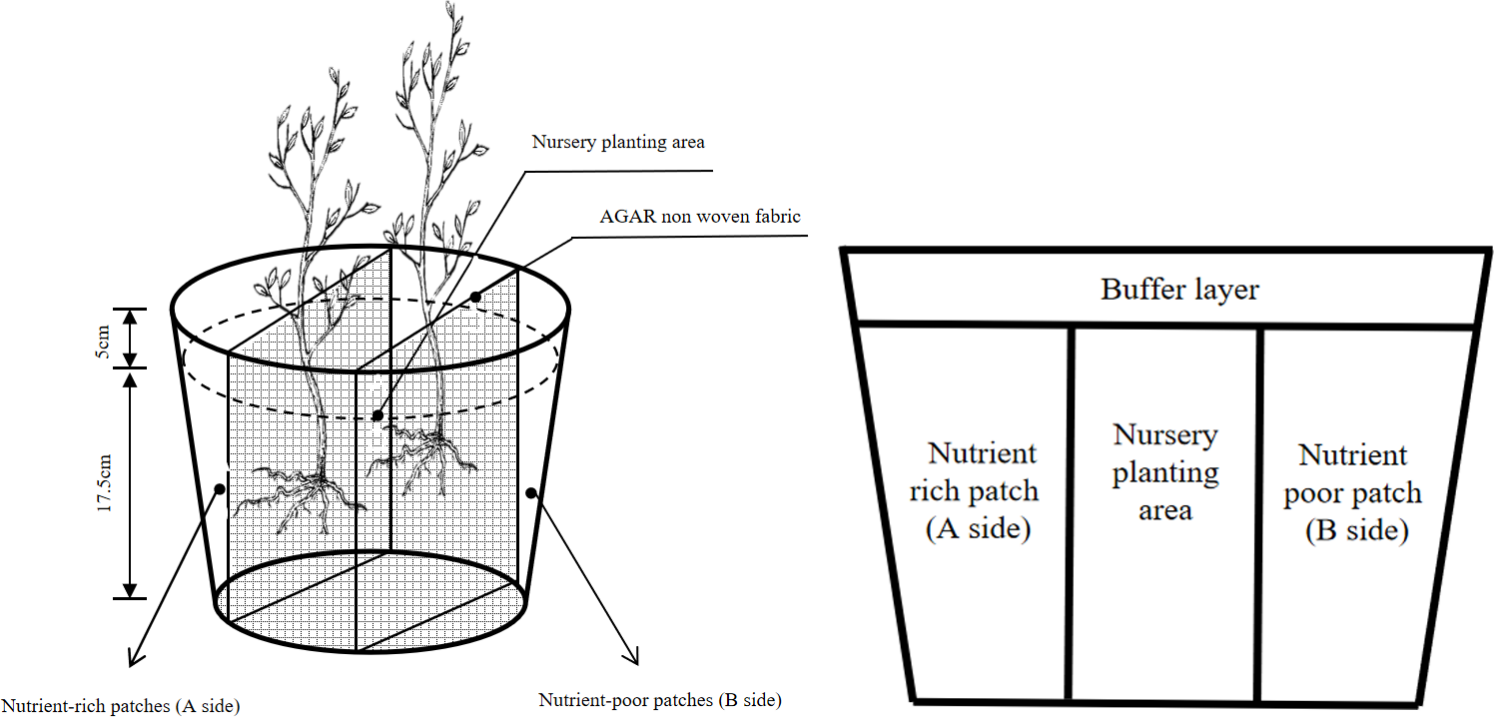
A front view of the pot container.

A 4×3 two-factor factorial design (consisting of four nutrient environments and three planting patterns) was adopted for this study. The *F. hodginsii* seedlings were planted in polyethylene pots with an upper inner diameter of 22.3 cm, a lower inner diameter of 15.5 cm, and a height of 20 cm. The experiment was started in early March 2022. First, the barren acidic red loam soil was transported from the hill behind Fujian Agriculture and Forestry University to the greenhouse for processing. The soil was then disinfected with 0.5% potassium permanganate, covered, sealed with a plastic film, and exposed to the sun for one week for air drying before sieving (This measure is effective in preventing the occurrence of standing blight, stem blight, sudden collapse, and root rot in plants.). After sieving, the soil was mixed with perlite at a mass ratio of 3:1 and used as the matching substrate for creating the heterogeneous and homogeneous nutrient environments. The upper part of the container was a buffer layer, which consisted of soil in the top 4 cm, and the bottom layer of soil that was filled to a depth of 16 cm was divided into three parts, namely, the nutrient-rich patch (A side), the nutrient-poor patch (B side), and the middle nursery planting area (**Figure 1**). (The nursery planting area is the initial growing area of the seedlings, in which the soil is potting substrate soil without any added fertilizer.). The two nutrient patches had the same volume and were separated from the nursery planting area by a non-woven fabric coated with agar. The agar-coated non-woven material was intended to prevent nutrient runoff and loss between the two plots, which would affect the test results while still ensuring that the root system of *F. hodginsii* seedlings can penetrate smoothly; this design was utilized as a convenient way to observe the growth of the root system in the later stage. Each pot was filled with around 4.5 kg of soil.

Nutrient formulations for heterogeneous/homogeneous environments were based on the cultivation of *F. hodginsii* by previous researchers. As there are fewer relevant studies on *F. hodginsii*, and only on N heterogeneity (Rong et al., 2020), based on the studies on nutrient heterogeneity in *Pinus massoniana* and *Cunninghamia lanceolata* (Wang et al., 2007; Ma et al., 2010) and the ratio of N, P, and K fertilization (Mou et al., 1997), it was concluded that the concentrations of N, P, and K in the fertilization of *F. hodginsii* should not be more than 110 mg·kg^-1^, 300 mg·kg^-1^, and 190 mg·kg^-1^, respectively. According to the nutrient ratios proposed by Mou et al. (1997) the nutrient ratio for N:P:K was 2: 5:3.

Therefore, when constructing homogeneous nutrient patches, the nutrient patches on both sides of the setup were established to have N, P, and K concentrations of 50, 125, and 75 mg·kg^-1^, respectively. One kilogram of the substrate was mixed with 0.1087 g of urea (N 46%), 1.0081 g of calcium superphosphate (P_2_O_5_ 16%), and 0.1433 g of potassium chloride (K_2_O 60%) on both sides of the container. To construct heterogeneous nutrient environments for different nutrient elements, one side of each container was filled with enriched soil for the corresponding nutrient element, such that twice the fertilizer amount corresponding to that element was applied so that the corresponding element content would be present at twice the concentration of that of the homogeneous environments, while the depleted environment would not have the corresponding nutrient element applied. For example, in the N heterogeneous nutrient environments, the nutrient-rich patches contained twice the amount of urea per kilogram of soil (0.2174 g), whereas the nutrient-poor patches on the other side of the barrier had no urea applied to ensure that the total amount of N, P, and K nutrients added to the heterogeneous and homogeneous nutrient environments were the same. The specific fertilization regime is shown in **Table 1**. In treatments with different heterogeneous nutrient environments, the A side represents the nutrient-rich side of the corresponding nutrient element in the heterogeneous environment, and the B side represents the nutrient-rich side. In treatments with a homogeneous nutrient environment, the contents of each nutrient element on both sides of the barrier were the same.

**Table 1:** Concentrations of N, P, and K in the heterogeneous (HET) and homogeneous nutrient patches.

Nutrient patch	Heterogeneous nutrient patch						Homogeneous nutrient patches					
	A (Nutrient-rich patches)			B (Nutrient-poor patches)			A			B		
	N	P	K	N	P	K	N	P	K	N	P	K
HET-N	100	125	75	0	125	75	50	125	75	50	125	75
HET-P	50	250	75	50	0	75						
HET-K	50	125	150	50	125	0						

Additionally, three planting patterns were adopted in the heterogeneous and homogeneous nutrient environments, including (1) the single-plant pattern, (2) the *F. hodginsii*–*F. hodginsii* planting pattern providing a conspecific neighbor, and (3) the *F. hodginsii*-*Cunninghamia lanceolata* planting pattern providing a heterospecific neighbor. (*Cunninghamia lanceolata* as the most common and suitable hybrid species for *F. hodginsii* has been confirmed by most research institutes, so we chose to use a mixture of *Cunninghamia lanceolata* and *F. hodginsii* seedlings to simulate interspecific competition.) One *F. hodginsii* seedling was planted in the middle of each pot in the single-plant pattern. In the conspecific neighbor pattern, two *F. hodginsii* seedlings were planted at two opposite points on the two sides of the midline, 5 cm away from the midpoint of the midline. In the heterospecific neighbor, one *F. hodginsii* seedling and one *Cunninghamia lanceolata* seedling were planted on the two sides of the midpoint of the midline. The *Cunninghamia lanceolata* seedlings were high-quality one-year-old seedlings obtained from the Yangkou State-owned Forest Farm of Fujian Province, China. The planting depths were about 5 cm. Each treatment consisted of 15 pots, yielding 180 pots in total. About 100 mL of purified water was applied daily per pot. Considering the long growth cycle, fertilization was conducted again in September 2022 and February 2023 to maintain the nutrient environments. The nutrient formulae and fertilization rates were the same as those in the first fertilization during planting.

### 2.4 Determination of the indicators

The plants were harvested in April 2023, and 10 normally growing plants were selected from each treatment. Each pot was cut in half with a blade, and each entire seedling was removed. The soil attached to the roots was washed away with tap water, and distilled water was used to further clean the roots. The water on the root surface was wiped dry with absorbent paper. The roots on both sides of the pot were tied with thin string and labeled as corresponding to rich- or poor-nutrient patches. After labeling, the non-woven fabric was cut open, and the complete root tissue was preserved as much as possible. The *F*. *hodginsii* seedlings with cleaned roots were taken back to the laboratory in self-sealing bags, and the root morphological characteristics parameters of each treatment were analyzed quantitatively using a WinRHIZO ProSTD1600+ image analysis system (Regent Instruments, Quebec, Canada). The total root length, total root surface area, total root volume, and average root diameter on both sides of the lower part of the pot were measured for all root tissue except for the main root.

The anatomical structure of the fine roots on both sides of the labeled *F*. *hodginsii* seedlings was observed and determined. Fifteen root tips of each treated seedling root sample were collected and soaked in glass vials containing FAA (formaldehyde, acetic acid, and ethanol) and then stored in ice bags to keep them cold (Pregitzer, 2002). The collected fine root samples were preprocessed, and paraffin sections were prepared through ethanol dehydration with a volume fraction of 60%, paraffin embedding, sectioning, and toluidine blue staining. Fine root sections of *C. lanceolata* from different sources were observed using a microscope (Nikon Eclipse; Nikon, Tokyo, Japan). In this process, relatively complete transverse sections within the field of view were selected, and the fine root diameter, vascular bundle diameter, cortical thickness, and vessel diameter were measured using NIS-Element AR software (Nikon). In addition, the ratio of the vascular bundle diameter to the root diameter was calculated.

The complete root system from each treatment was placed in an oven, desiccated at 105°C for 30 min, and then dried at 80°C to a constant weight. The biomass of root system of *F*. *hodginsii* seedlings in each treatment was measured. Finally, the biomass of seedlings in nutrient-poor and nutrient-rich patches was measured separately for the heterogeneous environments. Four parameters, namely specific root length、specific root surface area、sensitivity and foraging precision, were used to demonstrate the root morphological plasticity of plants in heterogeneous nutrient environments.

Specific root length is the sum of the lengths of plant roots per unit biomass; the higher the specific root length, the finer the diameter of the root system (Giuggiola et al., 2018). Specific root length is expressed as the ratio of the length of the root system to its dry mass.

Specific root surface area is the surface area of the plant root system per unit area. Accordingly, specific root surface area is expressed as the ratio of root surface area to the dry matter accumulation of the root system.

Foraging precision refers to the ability of the root system to seek nutrient-rich patches (Farley and Fitter, 1999; Fransen et al., 1998; Robinson et al., 1999). The calculation method is as follows:


$$FP=\frac{R_{1}-R_{2}}{R_{t}}$$

*FP* indicates foraging precision, *R*_1_ represents root dry matter mass in nutrient-rich patches, *R*_2_ represents root dry matter mass in nutrient-poor patches, and *R*_t_ represents the total root dry matter mass.

Sensitivity refers to the ability of plant growth to respond to the spatial heterogeneity of available nutrients, which is expressed as the ratio of total dry matter mass in heterogeneous and homogeneous nutrient environments (Wijesinghe and Hutchings, 2001; Einsmann et al., 1999). The calculation is as follows:


$$S_{R}=\frac{T_{1}}{T_{2}}$$

*S*_R_ indicates root sensitivity, *T*_1_ represents total dry matter mass in heterogeneous nutrient environments, and *T*_2_ represents total dry matter mass in homogeneous nutrient environments.

### 2.5 Statistical analysis

The SPSS 22.0 software (IBM Corp., Armonk, NY, USA) was used for all statistical analyses. All data were analyzed by one-way analysis of variance (ANOVA). Tukey’s HSD method was used to determine whether there was a significant difference between the indicators under different treatments (α = 0.05). Finally, a principal component analysis (PCA) was used to determine the dominant factors and comprehensively rank the combinations of different environmental factors. All the figures were created using Origin 2022 (OriginLab, Northampton, MA, USA).

## 3 Results

### 3.1 Response of root biomass and patch-induced characteristics of the *F. hodginsii* seedlings to heterogeneous nutrient environments and planting patterns

**Figure 2:**
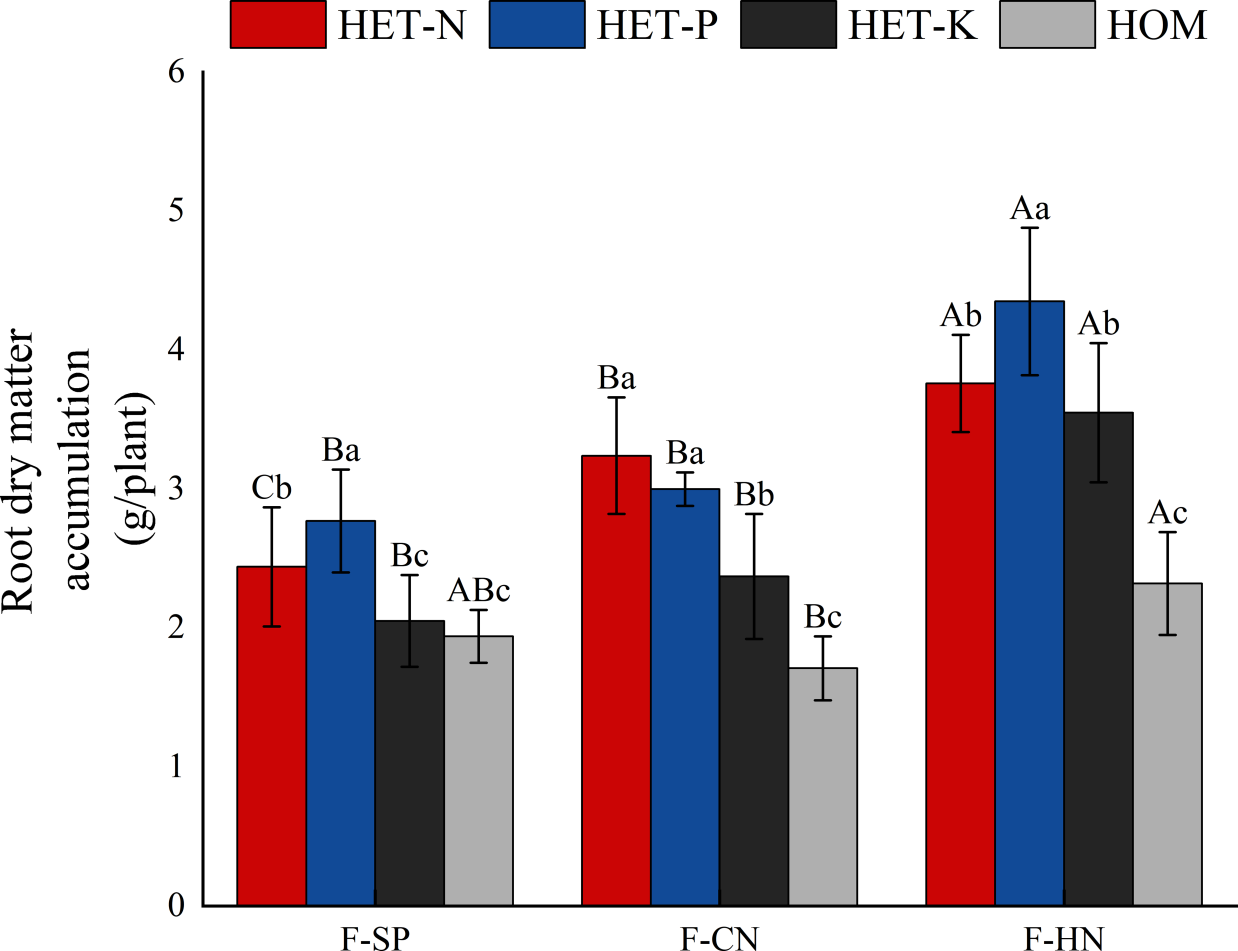
Differences in the accumulation of dry matter in *Fokienia hodginsii* seedling roots under different treatments. Different capital letters in the figure represent significant differences in the *F. hodginsii* indicators among different planting patterns under the same nutrient environment (*P<* 0.05). Different lowercase letters represent significant differences in indicators of *F. hodginsii* under different nutrient environments in the same planting pattern (*P<* 0.05). HET-N, N heterogeneous nutrient environment; HET-P, P heterogeneous nutrient environment; HET-K, K heterogeneous nutrient environment; HOM, homogeneous environment; F-SP, single-plant pattern; F-CN, conspecific neighbor; F-MP, heterospecific neighbor. The error bars represent the standard error.

Based on the impact of planting patterns on the dry matter mass of root system, *F. hodginsii* were more sensitive to heterogeneous nutrient environments under all three planting patterns (**Figure 2**). The average accumulation of dry matter in the root systems in the four nutrient environments with a heterospecific neighbor was 3.50 g, which was 35.5% higher than that with a conspecific neighbor. There was more dry matter in the *F. hodginsii* root system under the two competitive treatments than in the non-competitive treatment. The mass of matter increased with a heterospecific and conspecific neighbor in comparison with the single-plant treatment, respectively. This indicated that the competitive environment can promote the accumulation of dry matter in the root system of *F. hodginsii*. Regarding the impact of different heterogeneous nutrient environments on the root dry matter mass of *F. hodginsii*, the average root dry matter mass in the P heterogeneous nutrient environment was the highest value among all the heterogeneous nutrient environments. The average root dry matter mass values in the N, P, and K heterogeneous nutrient environments were 58.1%, 70.2%, and 34.0% higher than that in the homogeneous environment, respectively, indicating that *F. hodginsii* is a tree species with a strong ability to seek heterogeneously distributed nutrients.

As indicated in **Table 2**, the accumulation of dry matter in the *F. hodginsii* roots in nutrient-rich and nutrient-poor patches showed different trends in heterogeneous nutrient environments among different planting patterns. The dry matter mass of the root system performed more effectively in the nutrient-rich patches than in the nutrient-poor patches under all three heterogeneous nutrient environments. Among them, the dry matter of the nutrient-rich patches with a heterospecific neighbor in the P heterogeneous environment was the highest, and it was significantly higher than that of the corresponding nutrient-poor patches by 100.4% (*P<* 0.01). Moreover, under the heterospecific neighbor pattern, both nutrient-rich and nutrient-poor patches accumulated significantly more dry matter than those under conspecific neighbor and single-plant patterns. The dry matter of the *F. hodginsii* root system only showed significant differences between nutrient-rich and -poor patches in the heterospecific neighbor (*P<* 0.05). With a heterospecific neighbor in the nutrient-rich patch, this indicator was slightly higher than those in the conspecific neighbor and single-plant patterns, but not significantly (*P* > 0.05). For nutrient-poor patches, the values of the conspecific neighbor were higher than those of the heterospecific neighbor and single-plant patterns, but not significantly (*P* > 0.05). In the K heterogeneous nutrient environment, the nutrient-rich and -poor patches differed significantly in root system dry matter accumulation (*P<* 0.05) with a heterospecific neighbor, which was significantly higher than those of the conspecific neighbor and single-plant patterns in the nutrient-rich patches. The root system dry matter accumulation with a conspecific neighbor was slightly higher than that of the single-plant pattern, but the difference was not significant (*P* > 0.05).

**Table 2:** Differences in the accumulation of dry matter in the roots between the nutrient-rich and nutrient-poor patches of *F. hodginsii* under different planting patterns in heterogeneous nutrient environments (mean ± standard error).

Nutrient patch	Planting pattern	Root dry matter accumulation / g		
		HET-N	HET-P	HET-K
Nutrient-rich patches	A	1.041±0.205Aa	1.185±0.484Ca	1.083±0.300Ba
	B	1.148±0.384Aa	1.452±0.453Ba	1.096±0.233Ba
	C	1.249±0.378Aa	2.407±0.958Aa	1.290±0.477Aa
Nutrient-poor patches	A	0.869±0.188Aa	0.986±0.159Ba	0.801±0.102Ba
	B	0.964±0.285Aa	1.047±0.402Bb	0.807±0.188Ba
	C	0.901±0.313Ab	1.201±0.483Ab	0.908±0.269Ab

Different capital letters in the table indicate significant differences in F. hodginsii indicators among the different planting patterns under the same patch type (P< 0.05). Different lowercase letters indicate significant differences in the indicators among different types of patches of F. hodginsii under the same planting pattern (P< 0.05). HET-N, N heterogeneous nutrient environment; HET-P, P heterogeneous nutrient environment; HET-K, K heterogeneous nutrient environment; A, single-plant pattern; B, conspecific neighbor; C, heterospecific neighbor.

As shown in **Table 3**, specific root length of *F. hodginsii* in heterogeneous nutrient environments was lower than that in a homogeneous environment, with the lowest specific root length occurring in N heterogeneous environments, at 15%, 26%, and 45% lower than that in the P and K heterogeneous and homogeneous environments, respectively. The specific root lengths in the competitive and non-competitive treatments showed a clear regularity; the specific root surface area showed a trend similar to that of specific root length, with the specific root area of the homogeneous environment being higher than that of the heterogeneous environment, and that of the homogeneous environment being 68%, 52%, and 57% higher than those of the N, P, and K heterogeneous environments, respectively. The specific root area was higher with a conspecific neighbor than with a heterospecific neighbor or in the single-plant treatment. The sensitivity and foraging accuracy of *F. hodginsii* displayed significant differences under different planting patterns in the heterogeneous nutrient environments. In the N heterogeneous environment, the root system was more sensitive under competitive treatments than under the non-competitive treatment, and it was the highest with a conspecific neighbor. However, foraging accuracy was lower than that in the heterospecific neighbor and single-plant treatments. In the P heterogeneous nutrient environment, the root sensitivity and foraging accuracy under competition were higher than those without any competition and higher in the heterospecific neighbor pattern than in conspecific neighbor and single-plant patterns. The roots were more sensitive under competition than without competition in the K heterogeneous environment, whereas the foraging was more accurate without competition than under competition. This indicates that competition can effectively improve the sensitivity of the *F. hodginsii* root system, with the highest average value of root sensitivity in the P heterogeneous environment. The accuracy of the root foraging to planting patterns varied among different heterogeneous nutrient environments.

**Table 3:** Sensitivity and foraging accuracy of *F. hodginsii* to heterogeneous nutrient environments under different planting patterns.

Heterogeneous nutrient	Planting pattern	Specific root length/ (g.cm^-1^)	specific root surface area/ (cm^-2^.g^-1^)	Sensitivity	Foraging precision
HET-N	A	189.64	209.82	1.258	0.070
	B	181.86	216.89	1.895	0.057
	C	194.31	202.92	1.621	0.093
HET-P	A	189.93	205.84	1.428	0.072
	B	249.74	265.61	1.754	0.135
	C	211.61	221.62	1.875	0.277
HET-K	A	246.70	199.40	1.057	0.138
	B	264.93	262.77	1.386	0.122
	C	200.13	208.45	1.530	0.098
HOM	A	235.04	322.46		
	B	320.69	406.95		
	C	263.34	325.39		

Different capital letters in the table indicate significant differences in F. hodginsii indicators among the different planting patterns under the same patch type (P< 0.05). HET-N, N heterogeneous nutrient environment; HET-P, P heterogeneous nutrient environment; HET-K, K heterogeneous nutrient environment; HOM, homogeneous environment; A, single-plant pattern; B, conspecific neighbor; C, heterospecific neighbor.

### 3.2 Response of root morphology and patch-induced characteristics of the *F. hodginsii* seedlings to heterogeneous nutrient environments and planting patterns

**Figure 3:**
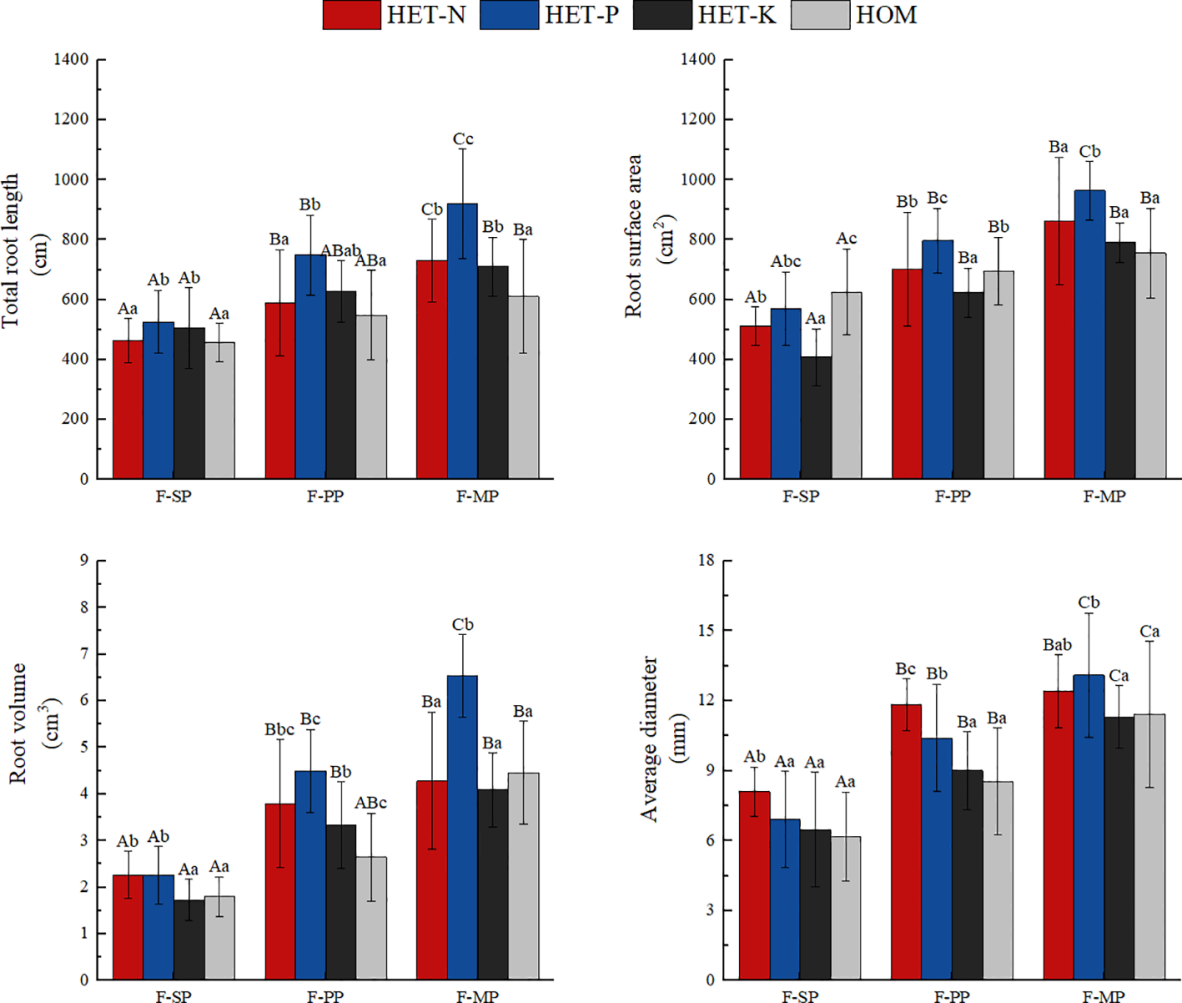
Differences in the root morphological parameters of the *Fokienia hodginsii* seedlings under different treatments. Different capital letters in the figure indicate significant differences in the *F. hodginsii* indicators among the different planting patterns under the same nutrient patch type (*P<* 0.05). Different lowercase letters indicate significant differences in indicators of *F. hodginsii* under the different nutrient patches in the same planting pattern (*P<* 0.05). HET-N, N heterogeneous nutrient environment; HET-P, P heterogeneous nutrient environment; HET-K, K heterogeneous nutrient environment; HOM, homogeneous environment; F-SP, single-plant pattern; F-CN, conspecific neighbor; F-MP, heterospecific neighbor. The error bars represent the standard error.

**Figure 4:**
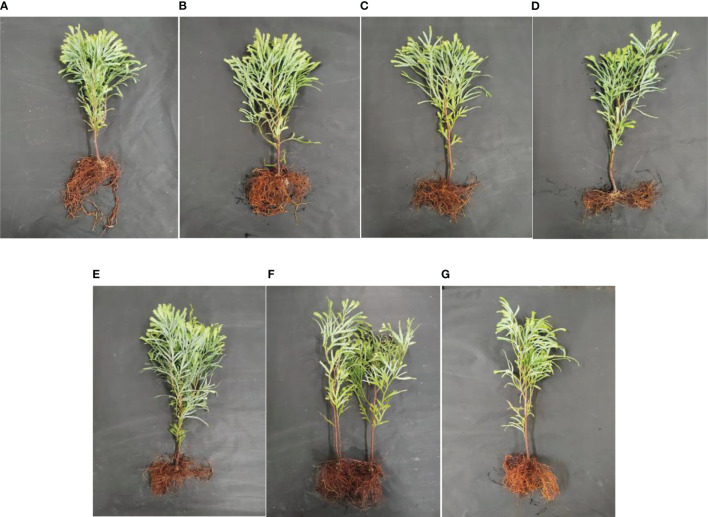
Growth differences of *Fokienia hodginsii* under different heterogeneous nutrient patches and planting patterns. Representative images of *F*. *hodginsii* under **(A)** the N heterogeneous environment; **(B)** the P heterogeneous environment; **(C)** the K heterogeneous environment; **(D)** the homogeneous environment; **(E)** the single-plant treatment; **(F)** the conspecific neighbor treatment; **(G)** the heterospecific neighbor treatment. The nutrient-rich patches are shown on the left side of each image, while the nutrient-poor patches are shown on the right side.

As shown in **Figure 3**, plants in the heterogeneous nutrient environments had longer total root systems than those in the homogeneous environments. Among the heterogeneous nutrient treatments, the average total root system in the P heterogeneous environment was the longest. This was 23.2%, 19.1%, and 35.9% higher than those in the N heterogenous, K heterogenous, and homogeneous environments, respectively. The P heterogeneous environment had the highest average total root surface area, which increased by 12.3% compared with that of the homogeneous environment. However, the total root surface area of the N and K heterogeneous environments was lower than that of the homogeneous environment. The total root volume under the heterogeneous environments was higher than that under the homogeneous environment, and those of N and P heterogenous environments were significantly increased by 16.2% and 49.4%, respectively. The total root volume under the K heterogeneous environment was slightly higher than those of the homogeneous environment, but the difference was not significant. In the N heterogeneous environment, the *F. hodginsii* root system had its largest average diameter, which was an increase of 6.2% compared with that in the P heterogeneous environment, but the difference was not significant. Average root diameter increased significantly compared with the K heterogeneous environment and homogeneous environment. This finding indicates that the P heterogeneous environment significantly altered the morphological structure parameters of the root system of *F. hodginsii* seedlings. **Figure 4** also demonstrates that the heterogeneous environments were generally conducive to the expansion of the root system and increased the elongation and quantity of the root system on the nutrient-poor side of the nutrient heterogeneous environment.

The root morphological structure of *F. hodginsii* under the competitive treatments was higher than that under the non-competitive treatments. Under the heterospecific neighbor treatment, the total length and average diameter of the root system across the four nutrient environments were consistent with significant increases of 52.4% and 74.3%, respectively, compared with that under the single-plant treatment. The heterospecific neighbor treatment also resulted in the highest total surface area and volume of roots. These results indicate that the *F. hodginsii* seedlings more robust root morphological parameters in the mixed pattern compared with the single-plant treatment. In the competitive treatments, the indicators of root morphological structure with a conspecific neighbor were all lower than those with a heterospecific neighbor; the total length, surface area, volume, and average diameter of the root system were 18.2%, 14.3%, 35.8%, and 21.3% lower than those with a heterospecific neighbor, respectively. **Figure 4** also indicates that with a heterospecific neighbor, *F. hodginsii* showed more expansion and growth of its root system compared with when it had a conspecific neighbor.

The results in **Table 4** demonstrate that under different planting patterns, the root morphological parameters of *F. hodginsii* in nutrient-rich and nutrient-poor patches exhibited different patterns. The total root length of *F. hodginsii* in the three heterogeneous nutrient environments demonstrated that the nutrient-rich patches promoted growth relative to the nutrient-poor patches. In the nutrient-rich patches, the root morphology under competition was significantly higher than that without competition, with plants under heterospecific competition outperforming those under conspecific competition, and all plants under competition outperforming plants under single-plant treatments. In nutrient-poor patches, the heterospecific neighbor treatment promoted root morphology parameters relative to the conspecific neighbor and single-plant treatments. The differences in the root morphology parameters between nutrient-rich and -poor patches were statistically significant in the P heterogeneous environment. Under competition in the three heterogeneous nutrient environments, the total root surface area in the nutrient-rich patches was significantly higher than that in the nutrient-poor patches. In contrast, the root surface area in the nutrient-rich patches was slightly higher than that in the nutrient-poor patches in the single-plant treatment, but the difference was not significant. In nutrient-rich patches, the competition promoted root morphology values that were significantly higher than those of the single-plant treatment, while those of the heterospecific neighbor treatment were higher than those of the conspecific neighbor across all three heterogeneous nutrient environments. The root surface area of the nutrient-poor patches varied among planting patterns, but the overall difference was not significant. The root volume under competition in the three heterogeneous nutrient environments was significantly higher in the nutrition-rich patches than that without competition. The root volume in the heterospecific neighbor treatment was significantly higher than that of the conspecific neighbor treatment in the N and P heterogeneous nutrient environments, but the difference was not significant in the K heterogeneous environment. The trend of the average root diameter in the different heterogeneous nutrient environments varied slightly from the other indicators. In the N and P heterogenous environments, average root diameter was higher in nutrient-rich patches than in nutrient-poor patches, and that under heterospecific competition was higher than that under conspecific competition, both of which were higher than that under the single-plant treatment. However, in the K heterogeneous nutrient environment, the average root diameter among the three planting patterns in the nutrient-poor patches was greater than that in the nutrient-rich patches, and there was a significant difference between the single-plant and conspecific neighbor treatments. This result suggested that competition can effectively promote the growth of root morphological structure of *F. hodginsii* seedlings, and most of the root morphology indicators were promoted in the nutrient-rich patches relative to the nutrient-poor patches in different heterogeneous environments.

**Table 4:** Differences in root parameters of *Fokienia hodginsii* between nutrient-rich and nutrient-poor patches in heterogeneous nutrient environments under different planting patterns.

Root morphology parameters	Nutrient patch	Planting pattern	Heterogeneous nutrient environments		
			HET-N	HET-P	HET-K
Total root length/(cm)	Nutrient-rich patches	A	267.605±54.255Ba	325.705±72.194Ca	278.149±77.503Ba
		B	429.665±98.360Aa	490.509±122.721Ba	360.669±68.223A
		C	500.754±81.721Aa	593.397±124.418Aa	413.444±73.580Aa
	Nutrient-poor patches	A	195.111±52.022Aa	215.406±45.493Bb	227.580±71.292Aa
		B	159.549±85.269Ab	248.708±72.111Bb	267.208±47.210Ab
		C	229.845±69.032Ab	327.108±87.572Ab	297.034±63.783Ab
Root surface area/(cm^2^)	Nutrient-rich patches	A	285.745±51.006Ba	313.414±61.203Ca	221.773±48.458Ba
		B	468.894±183.803Aa	537.088±167.506Ba	403.3695±52.074A
		C	533.298±185.912Aa	724.161±168.262Aa	492.088±106.648Aa
	Nutrient-poor patches	A	226.220±56.826Aa	256.756±76.532Aa	186.996±52.013Aa
		B	233.840±56.627Ab	259.732±57.708Ab	209.388±54.882Ab
		C	229.681±63.459Ab	239.874±57.989Ab	247.913±35.665Ab
Root volume/(cm^3^)	Nutrient-rich patches	A	2.428±0.723Ca	1.308±0.376Ca	1.003±0.345Aa
		B	4.916±1.651Ba	2.519±0.466Ba	2.827±0.805Aa
		C	5.723±1.664Aa	4.547±0.953Aa	3.268±0.984Aa
	Nutrient-poor patches	A	2.102±0.456Aa	0.953±0.329Bb	0.723±0.190Ab
		B	2.668±0.806Ab	1.975±0.468.Ab	0.500±0.115Bb
		C	2.841±0.504Ab	1.987±0.557Ab	0.821±0.147Bb
Average diameter/(mm)	Nutrient-rich patches	A	8.107±1.637Ba	7.321±2.079Ca	6.742±2.474Bb
		B	12.180±2.029Aa	10.791±2.525Ba	6.200±2.590Bb
		C	13.230±2.300A	14.070±3.251Aa	9.726±1.743Aa
	Nutrient-poor patches	A	8.102±1.277Ba	6.507±2.197Ca	8.292±1.918Ca
		B	11.458±1.511Aa	10.016±2.215Ba	12.181±1.367Aa
		C	11.564±1.670Ab	12.147±2.615Ab	10.414±1.769Ba

Note: Different capital letters in the table indicate significant differences in F. hodginsii indicators among the different planting patterns under the same patch type (P< 0.05). Different lowercase letters indicate significant differences in the indicators among different patches of F. hodginsii under the same planting pattern (P< 0.05). HET-N, N heterogeneous nutrient environment; HET-P, P heterogeneous nutrient environment; HET-K, K heterogeneous nutrient environment; A, single-plant pattern; B, conspecific neighbor; C, heterospecific neighbor.

### 3.3 Response of the fine root anatomical structure and patch-induced characteristics of *F. hodginsii* seedlings to heterogeneous nutrient environments and planting patterns

**Figure 5:**
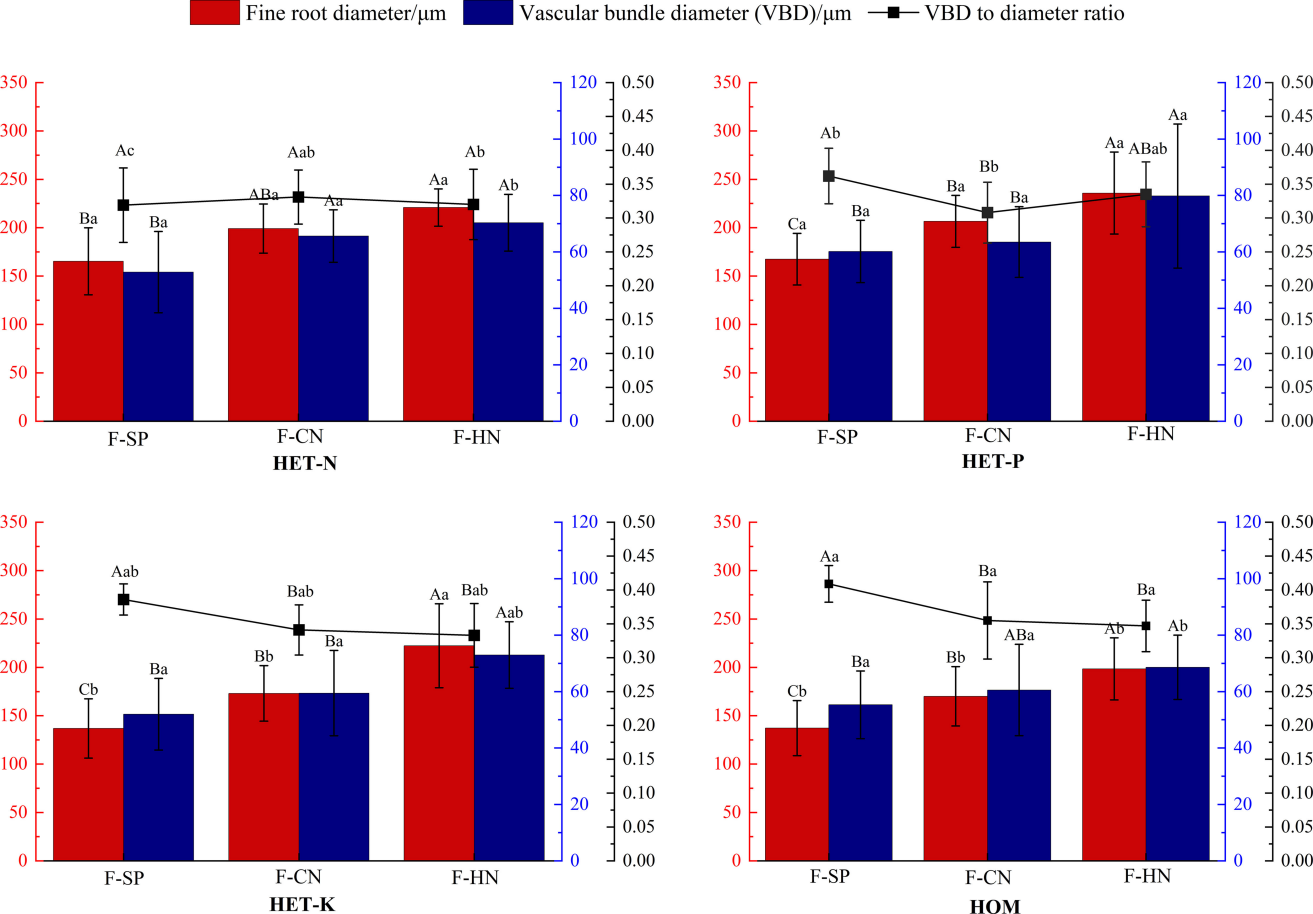
Differences in the fine root diameter, vascular bundle diameter, and the ratio of vascular bundle diameter to the root diameter of *Fokienia hodginsii* under different treatments. Different capital letters in the figure indicate significant differences in the *F. hodginsii* indicators among different planting patterns under the same nutrient patch type (*P<* 0.05). Different lowercase letters indicate significant differences in indicators of *F. hodginsii* under different nutrient patches in the same planting pattern (*P<* 0.05). HET-N, N heterogeneous nutrient environment; HET-P, P heterogeneous nutrient environment; HET-K, K heterogeneous nutrient environment; HOM, homogeneous environment; F-SP, single-plant pattern; F-CN, conspecific neighbor; F-MP, heterospecific neighbor. The error bars represent the standard error.

**Figure 6:**
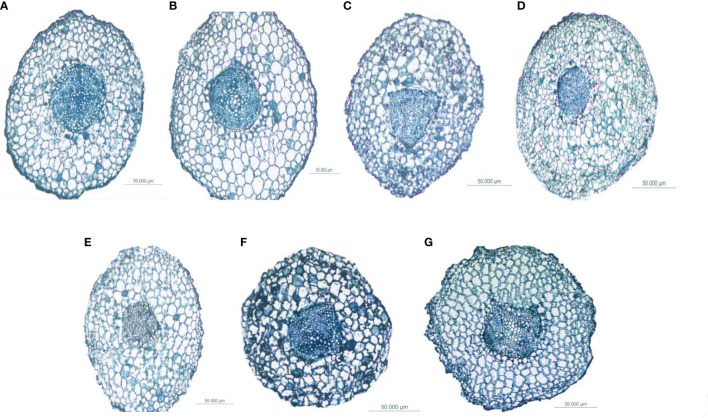
Differences in the anatomical structure of the fine roots of *Fokienia hodginsii* under different heterogeneous nutrient patches and planting patterns. Typical cross-sections of root vascular bundles of plants in **(A)** the N heterogeneous nutrient environment; **(B)** the P heterogeneous nutrient environment; **(C)** the K heterogeneous nutrient environment; **(D)** the homogeneous nutrient environment; **(E)** the single-plant planting pattern; **(F)** the conspecific neighbor planting pattern; **(G)** the heterospecific neighbor planting pattern.

As shown in **Figure 5**, the average diameter of the fine roots of *F. hodginsii* seedlings reached their highest value in the P heterogeneous environment, and this value was significantly higher, by 20.5%, compared with that of the homogeneous environment. The average fine root diameters of the N and K heterogeneous environment were also 15.7% and 5.3% higher, respectively. The diameter of the vascular bundle in the fine roots also reached its peak in the P heterogeneous environment and increased compared with that of the homogeneous environment. There was no significant difference between the N and K heterogeneous environments and homogeneous environment in terms of this parameter. In the homogeneous environment, there was a higher ratio of the vascular bundle diameter to the root diameter than those in all the heterogeneous nutrient environments. The N heterogeneous environment had the lowest vascular bundle diameter to root diameter ratio, which was 14.5% lower than that of the homogeneous environment. The vascular bundle diameter to root diameter ratio of the P and K heterogeneous environments was lower than that of homogeneous, respectively, but the differences were not significant. These results indicated that a heterogeneous nutrient environment can effectively increase the diameter of fine roots and vascular bundles of *F. hodginsii* seedlings compared with a homogeneous nutrient environment, but there was no significant effect on the ratio of vascular bundle diameter to root diameter (**Figure 6**).

The ratio of vascular bundle diameter to root diameter in *F. hodginsii* indicated that there were higher fine root and vascular bundle diameters under competition than without competition. Among the treatments, the fine roots under heterospecific neighbor treatment had a higher mean diameter among all the heterogeneous nutrient patches, which was 44.6% higher compared with that under the single-plant treatment. The fine root mean diameter in the conspecific neighbor treatment was lower than that in the heterospecific neighbor treatment, but was approximately 23.4% higher than that of the single-plant treatment. The heterospecific neighbor treatment also led to the highest mean diameter of vascular bundles in the fine roots, which was 17.1% and 32.4% higher compared with those of the conspecific neighbor and single-plant treatments, respectively. Under competition, the ratio of the vascular bundle diameter to the root diameter of *F. hodginsii* was lower than that without competition. This ratio under heterospecific neighbor treatment was slightly higher than that under conspecific neighbor treatment, but the difference was not significant. These data suggested that the competition can promote the growth of fine roots in *F. hodginsii* seedlings and increase the diameter of fine roots and vascular bundles, though competition reduced the ratio of vascular bundle diameter to root diameter to some degree (**Figure 6**).

**Figure 7:**
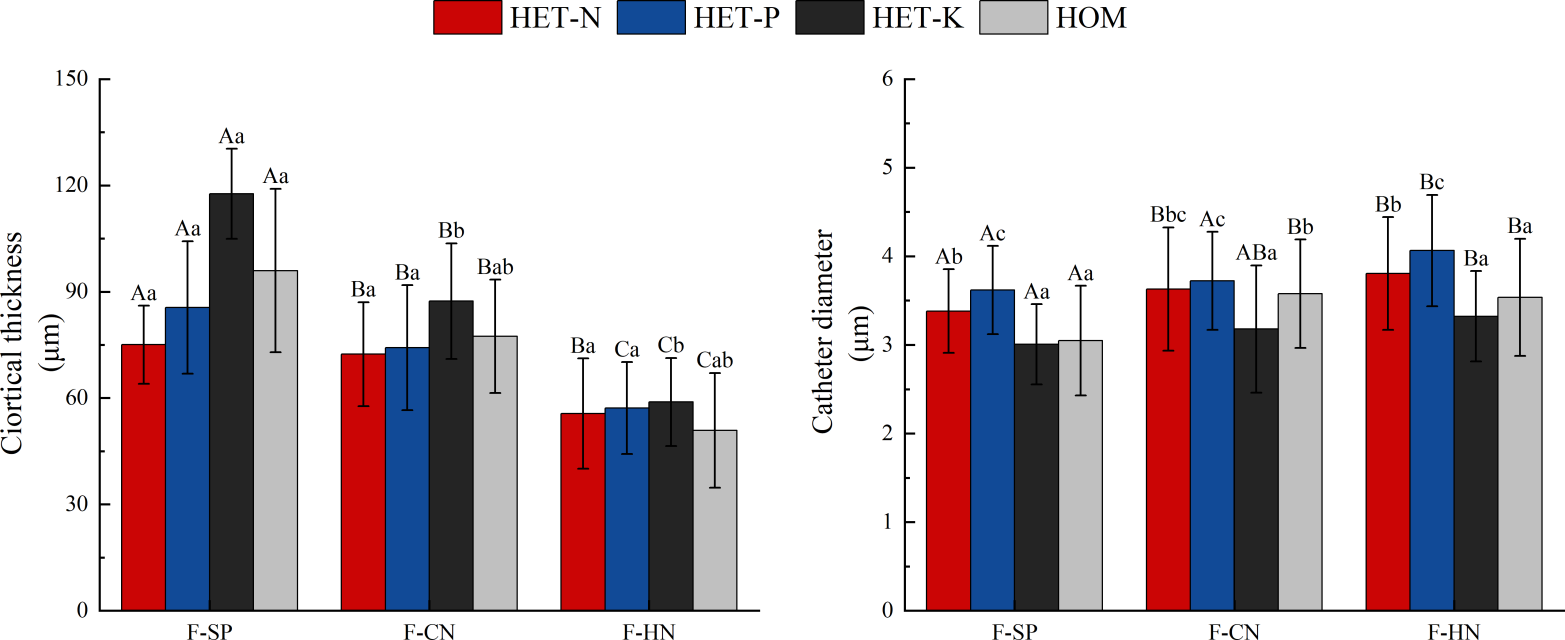
Differences in the cortex thickness and vessel diameter of the fine roots of *Fokienia hodginsii* under different treatments. Different capital letters in the figure indicate significant differences in the *F. hodginsii* indicators among different planting patterns under the same nutrient patch type (*P<* 0.05). Different lowercase letters indicate significant differences in indicators of *F. hodginsii* under different nutrient patches in the same planting pattern (*P<* 0.05). HET-N, N heterogeneous nutrient environment; HET-P, P heterogeneous nutrient environment; HET-K, K heterogeneous nutrient environment; HOM, homogeneous environment; F-SP, single-plant treatment; F-CN, conspecific neighbor; F-MP, heterospecific neighbor. The error bars represent the standard error.

As shown in **Figure 7**, the thickness of the fine root cortex of *F. hodginsii* seedlings reached a peak value in the K heterogeneous environment. This value was significantly higher, by 17.7%, compared with that of the homogeneous environment. However, the thickness of the fine root cortex in N and P heterogeneous environments was slightly lower than that in the homogeneous environment, but the difference was not significant. The diameter of the vessel showed the highest value, in the P heterogeneous environment followed by the N heterogeneous environment. The two indicators increased by 12.2% and 6.4%, respectively, compared with those in the homogeneous environment, while the vessel diameter in the K heterogeneous environment was lower than that in the homogeneous environment. Among planting patterns, the thickness of fine root cortex and vessel diameter showed opposite trends. The thickness of fine root cortex under competition was lower than that in without competition with a cortex thickness under the single-plant treatment. This value was significantly higher, by 20.2% and 68.1%, compared with those under conspecific neighbor and heterospecific neighbor treatments, respectively. In the heterospecific neighbor treatment, the vessel diameter was higher than that in the homogeneous environment, whereas the average diameter of vessel in conspecific neighbor was an increase of 8.1% compared with that in the homogeneous environment (**Figure 6**).

The results in **Table 5** show that under different planting patterns, the anatomical structure and various indicators of *F. hodginsii* in the nutrient-rich and nutrient-poor patches exhibited different patterns in heterogeneous nutrient environments. The fine root diameter and vascular bundle diameter of *F. hodginsii* in the three heterogeneous nutrient environments in nutrient-rich patches were larger than those of the nutrient-poor patches. The fine root diameter showed a significantly higher value under competition than without competition in both nutrient-rich and nutrient-poor patches. The vascular bundle diameter in the heterospecific neighbor treatment nutrient-rich patches was significantly higher than that in under the conspecific neighbor and single-plant treatments. In nutrient-poor patches, the values under the conspecific neighbor treatment in the N heterogeneous environment were slightly higher than those under the heterospecific neighbor treatment, but both were higher than those of the single-plant treatment. The P and K heterogeneous nutrient environments led to significantly higher values in the heterospecific neighbor treatment compared with conspecific neighbor and single-plant treatments, while the ratio of vascular bundle diameter to root diameter did not show significant changes between the different planting patterns and nutrient-rich and -poor patches. In the N heterogeneous environment, the cortical thickness of each planting pattern in the nutrient-rich patches was slightly lower than that in the nutrient-poor patches, but the difference was not significant. The single-plant treatment resulted in a value that was significantly higher than that observed under competition, but the difference between heterospecific neighbor and conspecific neighbor values was not significant. In the K heterogeneous environment, the cortical thickness of each planting pattern in the nutrient-rich patches was significantly lower than that in the nutrient-poor patches, while the single-plant treatment yielded a significantly higher value than that under competition. In addition, the cortical thickness of the conspecific neighbor treatments was significantly higher than that of heterospecific neighbor. No obvious differences in cortical thickness were observed in the P heterogeneous environment. Vessel diameters in the nutrient-rich and -poor patches under competition were significantly higher than those without competition, and the vessel diameters under the heterospecific neighbor treatment were higher than those under the conspecific neighbor treatment. Thus, competition promoted most of the indicators of fine root anatomical structure of *F. hodginsii* seedlings, and most of the indicators of fine root anatomical structure under the nutrient-rich patches in the different heterogeneous environments were greater than those under the nutrient-poor patches.

**Table 5:** Differences in the anatomical structure between nutrient-rich and nutrient-poor patches of *Fokienia hodginsii* under different planting patterns in heterogeneous nutrient environments (mean ± standard error).

Fine root anatomy	Nutrient patch	Planting pattern	Heterogeneous nutrient		
			HET-N	HET-P	HET-K
Fine root diameter /um	Nutrient-rich patches	A	176.543±36.904Ba	172.276±25.798Ba	147.293±30.443Ca
		B	204.241±22.461ABa	216.320±31.215ABa	186.510±34.851Ba
		C	230.020±21.540Aa	242.775±41.629Aa	232.266±47.808Aa
	Nutrient-poor patches	A	154.040±32.337Ba	162.530±27.481Ca	126.412±30.965Ca
		B	193.886±28.400Aa	196.693±22.39Ba	159.726±22.555Bb
		C	211.679±17.134Aa	228.822±43.159Aa	212.525±38.944Aa
Vascular bundle diameter (VBD) /um	Nutrient-rich patches	A	57.510±16.867Ba	65.382±13.016Ba	50.998±10.189Ba
		B	65.573±6.969Ba	66.948±13.833Ba	65.925±15.260ABa
		C	79.272±7.471Aa	79.009±25.139Aa	77.205±12.384Aa
	Nutrient-poor patches	A	48.120±11.953Ba	54.864±9.067Bb	52.927±15.242Ba
		B	65.649±11.629Aa	60.012±11.188Ba	52.953±14.988Bb
		C	61.380±12.646Ab	80.564±25.941Aa	68.701±11.195Aa
VBD to diameter ratio	Nutrient-rich patches	A	0.323±0.057Ba	0.382±0.064Aa	0.352±0.061Ab
		B	0.323±0.036Bb	0.309±0.042Ba	0.353±0.048Aa
		C	0.346±0.028Aa	0.320±0.059Bb	0.339±0.055Ba
	Nutrient-poor patches	A	0.315±0.053ABa	0.342±0.064Ab	0.420±0.064Aa
		B	0.339±0.044Aa	0.306±0.048Ba	0.329±0.065Bb
		C	0.294±0.075Bb	0.350±0.076Aa	0.327±0.038Ba
Ciortical thickness /um	Nutrient-rich patches	A	59.281±16.366Ba	59.055±14.039Ba	68.576±11.210Ca
		B	73.104±16.429Aa	80.406±16.995Ab	96.220±20.842Ba
		C	75.139±8.776Aa	68.023±18.276Bb	124.463±35.568Aa
	Nutrient-poor patches	A	51937±14.784Ba	55.269±11.923Ca	49.197±13.579Cb
		B	71.660±12.917Aa	91.802±21.877Aa	78.470±11.755Bb
		C	74960±13.243Aa	79.346±15.489Ba	110.802±21.843Ab
Catheter diameter /um	Nutrient-rich patches	A	3.561±0.427Ba	3.733±0.543Ba	3.252±0.473Ba
		B	3.617±0.531Ba	3.672±0.685Ba	3.337±0.927ABa
		C	4.049±0.643Aa	4.152±0.526Aa	3.461±0.666Aa
	Nutrient-poor patches	A	3.200±0.517Bb	3.505±0.451Cb	2.759±0.432Bb
		B	3.644±0.859Aa	3.773±0.418Ba	3.021±0.509Ab
		C	3.560±0.630Ab	3.975±0.732Ab	3.1850.351Ab

Different capital letters in the table indicate significant differences in F. hodginsii indicators among the different planting patterns under the same patch type (P< 0.05). Different lowercase letters indicate significant differences in the indicators among different patches of F. hodginsii under the same planting pattern (P< 0.05). HET-N, N heterogeneous nutrient environment; HET-P, P heterogeneous nutrient environment; HET-K, K heterogeneous nutrient environment; A, single-plant pattern; B, conspecific neighbor; C, heterospecific neighbor.

### 3.4 Comprehensive evaluation of the indicators of root morphological structure in *F. hodginsii*

A PCA was conducted on the root morphological structure of 10 F*. hodginsii* seedlings (**Table 6**). The percentage of the variance explained by the first principal component reached 77.789%, indicating it can basically explain the vast majority of the variation in the data. Therefore, the first principal component was used as a comprehensive evaluation index. The absolute values of the coefficients of fine root diameter, root volume, average diameter, and vascular bundle diameter in the first principal component were relatively large. This suggested that they are the most important physiological indicators that reflected the root morphological structure of *F. hodginsii* seedlings under different planting patterns and heterogeneous nutrient patches.

**Table 6:** Principal component analysis of the root morphological structure indicators of *Fokienia hodginsii* seedlings.

Parameters	Principal component
	1
Root dry matter accumulation	0.867
Total root length	0.932
Root surface area	0.927
Root volume	0.964
Average diameter	0.953
Fine root diameter	0.990
Vascular bundle diameter	0.951
VBD to diameter ratio	-0.694
Ciortical thickness	0.666
Catheter diameter	0.807
Eigenvalue	7.779
Contribution rate /%	77.789
Accumulative contribution 1%	77.789

The comprehensive evaluation score based on the eigenvalues of PCA was calculated (**Table 7**). The P heterogeneous environment and heterospecific neighbor, N heterogeneous environment and heterospecific neighbor, K heterogeneous environment and heterospecific neighbor, and P heterogeneous environment and conspecific neighbor treatment combinations had higher comprehensive evaluation scores.

**Table 7:** Comprehensive evaluation of the treatment effects of different nutrient environments and planting patterns on the root morphological structure indicators of *Fokienia hodginsii* seedlings.

Treatments (Planting patternxNutrient heterogeneous environments)	Comprehensive scores	Comprehensive rank
HET-N x F-SP	-1.71	10
HET-N x F-CN	0.74	6
HET-N x F-HN	2.14	2
HET-P x F-SP	-1.38	9
HET-P x F-CN	1.34	4
HET-P x F-HN	3.96	1
HET-K x F-SP	-3.14	12
HET-K x F-CN	-0.64	7
HET-K x F-HN	1.84	3
HOM x F-SP	-3.02	11
HOM x F-CN	-0.94	8
HOM x F-HN	0.82	5

HET-N, N heterogeneous nutrient environment; HET-P, P heterogeneous nutrient environment; HET-K, K heterogeneous nutrient environment; A, single-plant pattern; B, conspecific neighbor; C, heterospecific neighbor.

## 4 Discussion

The spatial distribution of forest soil nutrients results in gradients and patches, making the soil nutrient environment mostly heterogeneous (Niinemets and Valladares, 2004, Hu et al., 2004). The plant root system is often the most sensitive portion of plaints in the response to such heterogeneity, and a number of studies have shown that the root system of a larger number of plants in the nutrient-rich patches displays a general phenomenon of hyperplasia, which includes the elongation of the root system, an increase in the number of new roots, and a significant change in the morphology and structure of the root system (Pregitzer et al., 1993; Caldwell, 1994; Robinson, 1994; Gross et al., 1995). In the present study, compared with homogeneous environments, heterogeneous nutrient environments were able to significantly increase the accumulation of dry matter of the root system of *F. hodginsii* and significantly improve most of the indicators of root morphological structure. Among them, P patches had the highest dry matter mass, total length, total surface area, and total volume of the root system, which is similar to the results of some previous studies (Drew, 1975; Ma et al., 2010). This could be explained by the hypothesis that P is not highly mobile and is diffuse in the soil. Thus, a root system needs to proliferate extensively to acquire it. This induces the roots to grow into more soil to obtain sufficient P. As a result, the accumulation of dry matter and morphological structure of the *F. hodginsii* root system significantly increased in the P heterogeneous environment. The biomass and morphological structure indicators of the *F. hodginsii* root system in the K heterogeneous environment were lower than those in the N and P heterogeneous environments, and some morphological indicators were not significantly different from those of the homogeneous environment. This is also similar to the results of previous studies (Jin et al., 2011; Guo et al., 2023). This finding could be explained by the high content of K ions in the plant seedling stage that inhibited the absorption of other nutrients, which led to slow growth, inhibition of the accumulation of root dry matter, and root expansion.

In terms of the patchiness of the root system of *F. hodginsii* seedlings in different heterogeneous nutrient environments, the root systems all had more obvious topological behaviors in the nutrient-rich patches, and such topological behaviors were most significant in the P nutrient patches. In the P-rich patches, *F. hodginsii* seedlings showed a large proliferation of fibrous lateral roots, and root morphometric indexes, such as total root length, root surface area, and root volume, were significantly higher than those in homogeneous and other heterogeneous nutrient environments, indicating higher foraging accuracy and response sensitivity. There was a lower degree of lateral root proliferation than in the P-rich patches when the *F. hodginsii* seedlings encountered N-rich patches. In addition, the root scale, foraging accuracy, and response sensitivity were lower than those of the P-rich patches but higher than those of the homogeneous patches. This suggested that an N heterogeneous environment can also induce the lateral root proliferation of *F. hodginsii* to absorb soil nutrients and improve its ability to utilize nutrients. However, owing to the difficulty of the movement of P in the soil, the P patches are extremely stable, whereas N more easily diffuses in the soil than P (Drew et al., 1973). As *F. hodginsii* has shallow roots, it needs to develop a large number of roots in P-rich patches and increase the elongation and quantity of the root system on the side corresponding to the nutrient-rich patch to absorb sufficient P from the soil in a P heterogeneous environment. However, as N is easier to move and metabolize in the soil than P, the plant roots can obtain N without an extensively proliferated root system. Therefore, the expansion of the *F. hodginsii* root system under P patches was higher than that under N patches. When *F. hodginsii* was in a mixed planting pattern with *C. lanceolata*, its roots accumulated higher amounts of dry matter, foraged more accurately, and were more sensitive, which is similar to the results obtained by Bliss et al. (2002). This indicated that interspecific competition among neighboring plants partially promoted the root scale of *F. hodginsii* in heterogeneous nutrient environments and maintained high foraging accuracy to seek and utilize nutrients. As a result, the morphological structure and colonization ability of the root system improved in nutrient-rich patches, and the efficiency of nutrient absorption increased to adapt to the competitive environment.

Plants are inevitably influenced by neighboring plants as they explore nutrient patches, a process that has a significant impact on the plasticity of their foraging capacity and the efficient use of nutrient resources. In the present study, the dry matter mass and morphological structure of the root systems of *F. hodginsii* under competition were at a higher level than those in the single-plant treatment, and the dry matter accumulation, total length, total surface area, total volume, and average diameter of the root system with a heterospecific neighbor were higher than those with a conspecific neighbor. This is similar to the results of Yao et al. (2017), who suggested that the greater accumulation of dry matter in the root system of *F. hodginsii* under competition than without competition and the greater vigor of root systems of plants with a heterospecific neighbor than with a conspecific neighbor could be attributed to changes in plant behavior induced by neighboring plant competition, including the accumulation of root dry matter and the expansion of root morphology (Zhang et al., 2012; Zhang et al., 2016). These changes enable plants to obtain more nutrient resources under competition. The interaction between the roots varies for the same and different plant species (Cahill et al., 2010; Luo et al., 2012), and many studies attribute this phenomenon to the recognition between the self- and non-self-root systems. When the plant root system encounters a non-self-root system, it generates more roots and expands its root morphology compared with its encounters a self-root system to gain an advantage in the competition with other plants. When competing with the same plant species for limited space and resources, owing to the similarity in ecological niche and space and nutrients required, there is a tendency for the plants to produce inhibitory effects on each other, which results in a smaller root mass and reduced root morphology compared with that when mixtures are grown with other plant species. This is known as the “kin recognition effect,” in which root secretions are used as signaling substances to distinguish the genetic identity of competitors from neighboring plants and to respond accordingly (Karban and Shiojiri, 2010, Fang et al., 2011). According to Hamilton’s kin selection theory, plant root systems may tend to reduce foraging behavior when they encounter genetically similar or equivalent neighboring plants (Hamilton, 1964).

Since the anatomical characteristics of roots are stable and conservative, they are important indicators of plant organ-related functional traits and can precisely reflect the response of plants to environmental changes (Taylor and Peterson, 2000; Guo et al., 2004). In this study, the differences in fine root anatomical structure of *F. hodginsii* seedlings under different planting patterns and heterogeneous nutrient environments were analyzed. We found that a heterogeneous nutrient environment increases the average diameter of fine roots of *F. hodginsii* seedlings. In the P heterogeneous environment, the average diameter of fine roots and vascular bundle peaked and were significantly higher than those in the homogeneous environment followed by the N and K heterogeneous environments. There was a slightly higher ratio of vascular bundle diameter to root diameter in the homogeneous environment than in all the heterogeneous nutrient environments. This indicated that the heterogeneous nutrient environment was able to effectively improve the diameters of fine roots and vascular bundles in *F. hodginsii* seedlings compared with the homogeneous nutrient environment. The heterogenous nutrient environment slightly reduced the ratio of the vascular bundle diameter to root diameter, but this effect was not significant. This result suggested that N and P nutrient heterogeneous environments can induce the expansion and proliferation of the roots system of *F. hodginsii* seedlings, which requires greater diameters of fine root tips (or primary roots) and vascular bundles to increase the efficiency of absorbing nutrients and water in the soil. However, owing to the greater increase in fine root diameter in heterogeneous environments compared with the effect on the diameter of vascular bundle, this also led to a slightly lower ratio of the vascular bundle diameter to root diameter in *F. hodginsii* in nutrient heterogeneous environments compared with the homogeneous environment, which is also somewhat similar to the findings of previous research (Chen et al., 2010).

The thickness of fine root cortex in the *F. hodginsii* seedlings reached its peak in the K heterogeneous environment, and it was significantly higher than that in the homogeneous environment. In contrast, the thickness of fine root cortex in the N and P heterogeneous environments was slightly lower than that in the homogeneous environment. However, this difference was not significant. Seedlings in the P heterogeneous environment had the highest diameter of vessels followed by the N heterogeneous environment, while the diameter of the vessel in K heterogeneous environment was lower than that in the homogeneous environment. Previous studies have shown that plant fine roots with low cortical thickness, high vascular bundle diameter, and high vessel diameter can more effectively absorb and transport nutrients and water (Wells and Eissenstat, 2003). The fine roots of *F. hodginsii* seedlings had a greater vascular bundle diameter, greater vessel diameter, and lower cortical thickness in the N and P heterogeneous environments, which enabled their roots to absorb more sufficient nutrients to meet the requirement for root expansion and proliferation. However, there were relatively small differences in the vascular bundle diameter, vessel diameter, and cortical thickness in the homogeneous environments in the K heterogeneous environment. The plants absorbed nutrients and water less efficiently than in the N and P heterogeneous environments. This resulted in smaller differences in dry matter and root morphology between the *F. hodginsii* root system in the K heterogeneous environment and homogeneous environment compared with the N and P heterogeneous environments. The diameters of fine roots and vascular bundles under competition were both greater than those without competition, and the fine roots and vascular bundles in the heterospecific neighbor treatment had the highest average diameters among those in all the heterogeneous nutrient environments. However, the ratio of the vascular bundle diameter to the root diameter under competition was lower than that without competition, which indicated that competition can promote the growth of the fine roots of *F. hodginsii* seedlings. Thus, the interspecific competition between neighboring plants can significantly increase the diameter of fine roots and vascular bundles. Moreover, the fine root diameter expanded at a higher rate under competition than can the vascular bundle diameter. This resulted in a lower ratio of the vascular bundle diameter to root diameter under competition compared with the single-plant treatment. In addition, the cortical thickness of the fine roots under competition was significantly lower than that without competition, while the vessel diameter reached its peak in the heterospecific neighbor treatment. This indicated that different heterogeneous nutrient environments have different abilities to promote the cortical thickness and vessel diameter of fine roots, while competition can significantly increase the diameter of fine roots and reduce their cortical thickness.

Based on the PCA results, the absolute values of the coefficients of fine root diameter, root volume, average diameter, and vascular bundle diameter were significantly higher than those of the other indicators. This indicated that these root morphological structure indicators of the *F. hodginsii* seedlings were more sensitive to different planting patterns and heterogeneous nutrient patches, and thus, these indicators may better reflect the root morphological structure of *F. hodginsii* seedlings. The comprehensive evaluation score calculated by the PCA suggested that the treatment combinations with higher comprehensive evaluation scores were P heterogeneous environment and heterospecific neighbor, N heterogeneous environment and heterospecific neighbor, K heterogeneous environment and heterospecific neighbor, and P heterogeneous environment and conspecific neighbor treatment combinations.

## 5 Conclusions

In the P heterogeneous environment, the accumulation of dry matter, root morphology, and anatomical structure of the *F. hodginsii* seedlings were superior to those of the other nutrient heterogeneous environments. N and P nutrient patches can significantly improve the indicators of accumulation of dry matter, morphology, and anatomical structure of *F. hodginsii* seedlings compared with the homogeneous treatments. The K heterogeneous environment displayed weaker effects on the root morphology and development of *F. hodginsii* seedlings than the N and P patches, and some indicators were not significantly different from the homogeneous patches. The mixed planting pattern was more conducive to the expansion and development of the root morphology of *F. hodginsii* seedlings than the conspecific planting and single-plant planting patterns. The N, P, and K heterogeneous patches in the heterospecific planting pattern and the P heterogeneous patches in the conspecific planting pattern generated a higher evaluation score in the PCA. The results of this study suggest a novel approach for *F. hodginsii* seedling pre-cultivation and lineage selection, aiming at selecting *F. hodginsii* seedlings or lineages with high adaptability to heterogeneous nutrient environments and strong competitiveness to neighboring plants, so as to contribute to high-quality silviculture.

## Data availability statement

The original contributions presented in the study are included in the article/supplementary material. Further inquiries can be directed to the corresponding author.

## Author contributions

BL: Writing – original draft, Writing – review & editing. MD: Investigation, Writing – review & editing. YP: Data curation, Writing – review & editing. WC: Writing – review & editing. TH: Writing – review & editing. LC: Supervision, Writing – review & editing. YZ: Project administration, Writing – review & editing. JR: Validation, Writing – review & editing.
